# Neglected Zoonotic Diseases—The Long and Winding Road to Advocacy

**DOI:** 10.1371/journal.pntd.0002800

**Published:** 2014-06-05

**Authors:** Hayley E. Mableson, Anna Okello, Kim Picozzi, Susan Christina Welburn

**Affiliations:** Centre for Infectious Diseases and Division of Pathway Medicine, School of Biomedical Sciences, College of Medicine and Veterinary Medicine, The University of Edinburgh, Edinburgh, United Kingdom; Centers for Disease Control and Prevention, Kenya

## Abstract

**Background:**

Years of advocacy for the neglected tropical diseases (NTDs) have focused the world's attention on these diseases of the poor, resulting most recently in the 2012 “London Declaration” and the recent World Health Assembly Resolution WHA66.12 on NTDs in May 2013. Control of the endemic neglected zoonotic diseases (NZDs) would benefit from a similar campaign, which needs the support of a global community.

**Methodology/Principal Findings:**

The resolutions from all 66 World Health Assembly (WHA) meetings held between 1948 and 2013 were examined to determine how many contain a specific focus on any of the following eight NZDs as defined by the World Health Organisation (WHO): anthrax, bovine tuberculosis (TB), brucellosis, *Taenia solium* cysticercosis, cystic echinococcosis (hydatidosis), leishmaniasis, rabies, and zoonotic human African trypanosomiasis (HAT or sleeping sickness). Twenty-one resolutions adopted in the 16 assemblies between 1948 and 2013 targeted one or more of these eight NZDs, representing 4% of the total resolutions on infectious diseases passed to date. The 2013 adoption of Resolution WHA66.12 targeting all 17 NTDs marks a change in approach by the WHA. Whereas previous resolutions have targeted the NTDs as separate entities, the new approach of the combined resolution will help increase the overall momentum to target these ancient diseases as coendemic clusters in endemic countries. However, three major NZDs remain outside this recent resolution: anthrax, brucellosis, and bovine TB.

**Conclusions and Significance:**

The recent adoption of a specific resolution at the WHA in 2013 that emphasises a One Health approach for the successful control of 17 NTDs is a major development in advocacy. However, recognition of the importance of three major NZDs to public health in endemic countries—anthrax, brucellosis, and bovine tuberculosis—is still lacking despite being prioritised by the WHA as early as the 1950s. Global advocacy for control of the NZDs as a whole would similarly benefit from adoption of a One Health approach as is promoted for the NTDs under WHA66.12.

## Introduction

Control of zoonotic disease requires integrated action from both human and animal health sectors alongside support and consultation from other sectors or industries. The rapid response to recent zoonoses outbreaks, such as those of West Nile virus and monkeypox in the United States, Hendra virus in Australia, Nipah virus in Malaysia and Singapore, severe acute respiratory syndrome (SARS) in China and Canada, and highly pathogenic avian influenza (HPAI), demonstrates how the mobilisation of considerable political and financial support for the control of emerging diseases is possible on a regional—if not global—scale. These emerging and reemerging zoonoses would appear to be far from “neglected;” the Food and Agriculture Organisation of the United Nations (FAO)'s “Global Programme for Avian Influenza Control and Eradication” received a projected budget of US$882 million in 2006 [1]. HPAI in particular continues to attract a substantial amount of support [2], largely driven by “fearful” policy narratives concerning global health securitisation and pandemic preparedness [3].

In contrast to the emerging infectious diseases (EIDs), the group of often coexisting neglected tropical diseases (NTDs), found predominantly throughout the developing world, are estimated to kill around half a million people every year [4]–[6]. The official World Health Organisation (WHO) list of the 17 NTDs includes Buruli ulcer, Chagas disease, dengue, dracunculiasis (guinea-worm disease), echinococcus, food-borne disease, human African trypanosomiasis (HAT or sleeping sickness), leishmaniasis, leprosy, lymphatic filariasis, onchocerciasis (river blindness), rabies, schistosomiasis, soil-transmitted helminthases, taeniasis/cysticercosis, trachoma, and yaws (endemic treponematoses). The linkages between NTDs, poverty, and the Millennium Development Goals are clearly acknowledged in the literature, for example, the WHO's “Global Plan to Combat Neglected Tropical Diseases” [7]. In addition to this group of 17 NTDs, the WHO has identified a subgroup of eight endemic or “neglected zoonotic diseases” (NZDs): anthrax, bovine tuberculosis, brucellosis, *T. solium* cysticercosis, cystic echinococcosis (hydatidosis), leishmaniasis, rabies, and HAT [8]. These diseases are all common where poverty, reliance on livestock or wildlife for social and financial capital, poor resilience, and the close proximity of people and their animals favour transmission [8], [9]. Unlike the emerging zoonoses, the endemic zoonoses may truly be cast as “neglected” in terms of political profile and relative funding, presenting a greater challenge to researchers, policy makers, and human and animal health professionals working to control these diseases in many parts of the world. This historic lack of investment around NTDs, particularly the NZDs, has largely been attributed to difficulties in estimating their overall burden on society, with the lack of available or accessible diagnostics coupled with wider systemic causes of underreporting cited as difficulties in obtaining accurate evidence for policy [7]–[9].

Tackling the problem of “neglect” in relation to any disease requires high-level advocacy. A critical step in this process is to reach the World Health Assembly, the supreme decision-making body of the WHO [10]. Meeting annually since WHO's establishment in 1948, the Assembly is attended by representatives from all 194 WHO Member States. Subjects discussed at the WHA are brought forward from Executive Board meetings where resolutions to be taken to the WHA are proposed. Adopted resolutions provide an immediate indication of where WHO focus will lie in terms of policies and programmes that are planned or are taking place worldwide. In this paper, we review all resolutions between 1948 and 2013 for their reference to the eight WHO-defined NZDs that contribute to poverty and examine their recent pathways to advocacy.

## Methods

Resolutions from all 66 WHA meetings held between 1948 and 2013 were examined to determine those that include a specific focus on the eight major NZDs. WHA resolutions from 1948–1992 are published in three editions of the *Handbook of Resolutions and Decisions of the World Health Assembly and the Executive Board* (WHA 1973, 1985, and 1993) [11]–[13], and accepted resolutions are listed by health sector—for example, “Protection of Mental Health” or “Promotion of Environmental Health” [12]. All resolutions relating to infectious diseases were identified. Resolutions relevant to NZDs were selected from the annual lists of resolutions (see Table 1) [14]–[17]. WHA resolutions from 2000–2013 are published on the WHO website [18]. Recommendations and references from resolutions for the Special Programme for Research and Training in Tropical Diseases (TDR), which was established in 1975, were also included in this evaluation given that the TDR programme includes both HAT and leishmaniasis [19].

**Table 1:** 

Disease	Year	Resolution	Additional Mention	Important Points
Anthrax	2002	WHA55.16 (Bioterrorism)	1950 Expert Committee on Zoonoses	Advocacy from risk of bioterrorism and threat to trade rather than due to contribution to poverty.
Bovine Tuberculosis (BTB)	1950	WHA3.28	Expert Committee on Zoonoses (1950), Expert Committee on Tuberculosis (1950)	Significant progress between 1950 and 1958, particularly regarding eradication of infected cattle. No subsequent mention since 1958. BTB is excluded from Resolution WHA66.12.
Brucellosis	1948	WHA1.4	None	Despite expert committee recommendations for regional control centres, there has been no specific reference to brucellosis since 1950. Brucellosis is excluded from Resolution WHA66.12.
Cysticercosis *T. solium*	2013	WHA66.12	None	Highlighted in the WHO's “Global Plan to Combat Neglected Tropical Diseases 2008–2015” and the 2012 WHO NTD Roadmap.
Cystic Echinococcus (hydatidosis)	1950	WHA3.23	Expert Committee on Zoonoses (1950), WHA66.12 (2013)	
Leishmaniasis	2007	WHA60.13		Listed by the Expert Committee on Zoonoses (1950) as requiring additional study.
Rabies	1950	WHA3.20	Expert Committee on Zoonoses (1950), WHA5.60 (1952), WHA66.12 (2013)	Described as “one of the most dreaded diseases” in the first decade of the existence of WHO.
TDR Resolutions Including HAT and Leishmaniasis	1974	WHA27.52	WHA28.51(1975), WHA28.70 (1975), WHA28.71(1975), WHA29.71(1976), WHA30.42 (1977), WHA43.18 (1990)	
Human African Trypanosomiasis	1983	WHA36.31	WHA 50.36 (1997), WHA56.7 (2003), WHA57.2 (2004)	No resolutions (including WHA66.12) distinguish the HAT subspecies responsible for acute *T. b. rhodesiense* and chronic *T. b. gambiense*).
Neglected Tropical Diseases	2013	WHA66.12		Encompasses 17 WHO-listed NTDs including 5 NZDs. Excluded from WHA66.12 are the three bacterial NZDs (anthrax, BTB, and brucellosis)
Zoonoses	1969	WHA22.35	WHA31.48 (1978)	

## Results

### WHA Resolutions and the Neglected Zoonotic Diseases 1948–2013

Zoonotic diseases have been prioritised in WHO policy since its inception in 1948, with 15–20 zoonotic diseases considered of major global importance in the initial work of the organisation [20]. Rabies, brucellosis, hydatidosis, and bovine tuberculosis were specifically highlighted in the early years, with efforts made to standardise methods for managing these diseases in partnership with the FAO and other agencies [20]. Rabies and brucellosis were assigned expert committees that led to the formation of the WHO Expert Committee for Zoonoses (WHO ECZ) in 1950. The WHO ECZ held a number of joint meetings with FAO in subsequent years to make recommendations for zoonotic disease control, addressing in particular bovine TB, anthrax, and cystic echinococcus [21]–[23]. A joint FAO/WHO meeting in 1967 highlighted more than 150 zoonoses [22].

In addition to the WHO ECZ, 21 resolutions adopted in the 16 Assemblies held between 1948 and 2013 targeted one or more of the eight NZDs, representing 4% of the identified 385 resolutions adopted on infectious diseases during this time. Seven of these 21 resolutions involved the TDR, including two calling for the development of a specific programme to control tropical diseases (WHA27.52 and WHA28.51) and subsequent resolutions commending the work done on the programme (WHA43.18) [12], [13]. Resolution WHA66.12, adopted at the 66th WHA in May 2013, represented the first occasion that a specific resolution for the group of 17 NTDs had been made, incorporating five of the eight major NZDs: cystic echinococcus, *T. solium* cysticercosis, leishmaniasis, rabies, and HAT.

**i) Anthrax.** Anthrax was one of five diseases dealt with in detail at the inaugural meeting in 1950 of the Expert Committee on Zoonoses [21]. The disease was deemed a significant risk to public health and trade given the tenacity of anthrax spores over time, and this discussion resulted in the provision of guidelines for the handling of potentially contaminated products. While there has been no specific mention of anthrax as a zoonotic disease at the WHA, the disease is addressed by the recent Resolution WHA55.16 (2002), which concerns bioterrorism.

**ii) Bovine tuberculosis.** Bovine tuberculosis was also considered by the joint Expert Committees on Zoonoses and acknowledged at the fifth session of the Expert Committee on Tuberculosis in 1950. The committee recognised the public health importance of bovine tuberculosis, along with its role in the economic status and nutritional standards of endemic countries [24]. Resolution WHA3.28 promoted interagency collaboration and proposed technical assistance to those countries in which the disease was a problem [11]. Between the first and second of the Expert Committee reports, eradication of diseased cattle became an important objective in many countries, with considerable progress made between 1950 and 1958 [22]. However, there have been no further bovine-TB-specific resolutions, and bovine TB is not included in WHA66.12.

**iii) Brucellosis.** At the time of WHO inception, brucellosis was considered a disease of major public health importance due to the physical suffering and reduced work ability of those infected, along with the decreased productivity of diseased livestock [20]. An expert group on brucellosis was convened at the inaugural WHA in 1948, with the subsequent Resolution WHA1.4 in the same year calling for a world centre for brucellosis. A second Executive Board meeting in 1948 reviewed brucellosis [11] and requested that the WHO Director-General designate regional centres for control of the disease. However, there have been no further brucellosis-specific WHA resolutions, and the disease is not included in WHA66.12.

**iv)** ***T. solium*** **cysticercosis.** Mention is made to cysticercosis for the first time in a resolution in 2013 under WHA66.12 as one of the 17 listed NTDs. The disease had been previously highlighted in the WHO's “Global Plan to Combat Neglected Tropical Diseases 2008–2015” as a “tool-ready” disease for which control options exist [7] and mentioned in specific action points of the 2012 WHO NTD Roadmap as a disease for which validated strategies for control and elimination should be available by 2015, enabling interventions to be scaled up in selected countries by 2020 [25].

**v) Cystic echinococcus (hydatidosis).** Cystic echinococcus was among the five diseases considered at the first Joint Expert Committee on Zoonoses in 1950 and recognised as a worldwide public health problem. Subsequently, resolution WHA3.23 in 1950 recognised the role of echinococcus in both human infection and as a cause of reductions in the food supply chain, requesting that the WHO Director-General lend assistance to countries for research and eradication [11]. The committee outlined control efforts aimed at reducing the disease in its domestic canine reservoir [21]. Hydatidosis has not been included in any subsequent resolution until its inclusion under the NTDs in WHA66.12.

**vi) Human African trypanosomiasis (zoonotic sleeping sickness).** Despite human African trypanosomiasis (HAT) being one of the diseases for which a study was initiated by the first international office of public health, which was created in 1907 [20], HAT was not represented in the 1950 Expert Committee on Zoonoses [21] and was not included in any WHA resolutions until it was named as one of the major parasitic diseases in WHA27.52 [12]. Four subsequent resolutions refer to HAT, including the 2013 NTD Resolution WHA66.12 [12], [14]–[16]. Despite the fact that HAT comprises two distinct diseases (chronic Gambian and acute Rhodesian sleeping sickness) that demand different control approaches, they are not distinguished in the currently adopted resolutions nor within any TDR directive.

**vii) Leishmaniasis.** Leishmaniasis was included in the first phase of work of the TDR but was not specifically addressed at the WHA until 2007, despite the 1950 Expert Committee listing this as one of the diseases that required further study. The passing of WHA60.13 in 2007 was an important step in advocacy for this disease, acknowledging leishmaniasis as “one of the most neglected tropical diseases.” Countries where it is a public-health problem have been urged to reinforce efforts for control and strengthen existing epidemiological data [17].

**viii) Rabies.** Due to fatality upon the appearance of its clinical signs, rabies was described as “one of the most dreaded diseases” in “The First Ten Years of the World Health Organisation” [20]. Its importance was related to the number of people that had to undergo rabies treatment each year, despite the low number of human deaths recorded [20]. Rabies was addressed at the first WHA, but no resolutions were adopted. Six months later, the Executive Board recommended an expert committee review the methods of treatment and control of rabies, leading to the adoption of Resolution WHA3.20 in 1950 [11]. Following the first session of the Joint WHO/FAO Expert Group on Zoonoses in 1950, further research by WHO and FAO regarding global burden and control by international regulation was recommended [21]. WHA5.60 was adopted in 1952; however, it was decided that an expert group on rabies should only be convened if an advance in research warrants it [11]. There were no subsequent resolutions that specifically addressed rabies until WHA66.12 included this disease within the group of 17 targeted NTDs.

### Resolution on Neglected Tropical Diseases

The adoption of Resolution WHA66.12 in May 2013 follows the London Declaration on NTDs in 2012 [26] and is a landmark for advocacy for the NTDs and NZDs; it was described as a “rags-to-riches story” by the WHO Director-General, Margaret Chan, at the 65th WHA (2012) [10]. Lorenzo Savioli, director of the WHO NTD department, declared the Resolution “an historic step in accelerating the fight against these diseases of poverty” [27]. WHA66.12 recognises previous successes for NTD control and recommends advocacy for long-term NTD financing that complements and sustains national commitment towards the targets set in the 2012 NTD Roadmap. It also encourages coordination with Veterinary Public Health actors under a One Health approach; given the animal and environmental contributions to transmission and control, this is particularly critical for sustainable control programmes for the NZDs. More importantly, the adoption of a resolution that targets all 17 NTDs as a group marks a significant shift in policy by the WHA. While resolutions previously targeted specific NTDs separately, sufficient momentum can now be built towards control and eradication of these diseases as a group. Significantly, the cluster of bacterial NZDs that present a significant burden to rural populations in endemic countries—anthrax, brucellosis, and bovine TB—are not included in Resolution WHA66.12 given that they are omitted from the “17 designated NTDs.” WHA66.12 does, however, urge the expansion of interventions to reach targets set out in WHO's “Global Plan to Combat Neglected Tropical Diseases 2008–2015,” within which anthrax and brucellosis are listed as “tool-deficient” diseases for which better control methods need to be developed.

### Pathways to Advocacy for the NZDs

The NZDs received specific mention in the WHO report “Working to Overcome the Global Impact of Neglected Tropical Diseases,” launched by the Director-General, Dr. Margaret Chan, on 14 October 2010. This report acknowledged their importance, flagging veterinary public health as one of five public-health strategies for the prevention and control of NTDs. The announcement by the United Kingdom (UK)'s Minister for International Development of a US$785 million four-year “landmark commitment” to address neglected tropical diseases in Africa in January 2012 firmly recognises the importance of NTDs and NZDs.

The subsequent WHO Roadmap for NTDs, “Accelerating Work to Overcome the Impact of Neglected Tropical Diseases” (2012), acknowledges that control of NZDs is cost-effective and can secure livelihoods as well as save lives [25]. The Roadmap comments on the progress that has been made through a series of NZD meetings, setting out goals for the elimination and eradication of certain NTDs by 2015 and 2020 using previous WHA resolutions as a guide [25]. Many of the proposed strategies for NTD control focus on preventive chemotherapy in humans, for example, administration of antihelmintic drugs to address onchocerciasis, schistosomiasis, and the soil-transmitted helminths. In contrast, preventative chemotherapy in humans may not be the most appropriate or cost-effective control strategy for many NZDs given the huge role of the animal or vector reservoirs in many transmission cycles. There is therefore a chance that diseases such as HAT, rabies, and the bacterial zoonoses could potentially be overlooked in the Roadmap.

Sustained institutional support for NZDs has also been promoted by a series of high-profile meetings for advocacy since 2005, led by WHO. This meeting series, supported by the UK Department of International Development (DFID), FAO, the World Organisation for Animal Health (OIE), and the European Union amongst others, highlights the intersectorial action required for the successful control of these diseases [8], [28], [29]. The inaugural NZD advocacy meeting, entitled *The Control of Neglected Zoonoses: A Route to Poverty Alleviation* and held in Geneva in 2005, had the objective “to bring together groups that would not ordinarily meet to address a common problem of interest” in keeping with the One Health approach [8]. The meeting focused on reasons why the poor suffer disproportionately from NZDs and made recommendations for research and collaboration. The advantages of joint human-animal health systems was considered key for successful NZD control, leading to an overarching recommendation to “work towards the concept of ‘One Health’” [8].

The second meeting in Nairobi in 2009, entitled *The Integrated Control of Neglected Zoonoses in Africa: Applying the One Health Concept* [28], connected a range of additional stakeholders to develop a strategic framework for the control of NZDs in keeping with the action points arising from the 2005 Geneva meeting. Recommendations were made for action plans for the NZDs at national, regional, and global levels to assess the burden of neglected zoonotic diseases in Africa and beyond and provide a framework for future control.

A third international NZD meeting, “*The Control of Neglected Zoonotic Diseases: Community-based Interventions for the Prevention and Control*” [29], focused on community-based approaches for NZD control from which four themes emerged: the impact of underestimating burden, the importance of community engagement, the control of animal reservoirs, and innovative approaches to advocacy for NZDs. Based around these themes, the consultation recommended the creation of a roadmap to combat NZDs where they are most prevalent [29].

The consistent message to promote NZD control under One Health has also been adopted by the Tripartite. In 2011, a High-Level Technical Meeting (HLTM) was convened by WHO/FAO/OIE to discuss priorities at the human-animal-ecosystem interface within the One Health vision [30]. The HLTM highlighted rabies as a priority, indicating that advocacy for NZDs is steadily improving. Interaction between stakeholders encouraged by the Tripartite and collaboration in control efforts may pave the way for zoonotic diseases to move higher up the agenda of disease control and international health and to shed their “neglected” sobriquet.

### Conclusion

There is a perception that the NTDs and NZDs have been historically “neglected” by decision makers in terms of their political profile and allocation of funding for research. There are a number of reasons for such neglect, largely driven by the fact that their burden is often solely found in developing countries, where the majority of effort in recent years has focused on HIV/AIDs, tuberculosis, and malaria. However, it is also difficult to gather information about the extent and impact of these diseases of the poor due to ineffective diagnostic capacity and poor health delivery systems that result in underestimation of disease burden. Unless a problem can be quantified, it is difficult to argue for funding and attention by policy makers.

This review has attempted to examine the extent of high-level political advocacy that has taken place for the NZDs since the inception of the WHO in 1948. By examining the various resolutions and references to the eight WHO-listed NZDs throughout the 16 WHA meetings since 1948, it is clear that these diseases have indeed been neglected in recent years in terms of higher policy dialogue at the WHA. A key step in the advocacy process is to achieve the passing of a WHA resolution for the disease(s) in question. In May 2013, Resolution WHA66.12 attempted to address this imbalance for the NTDs. However, three NZDs were omitted from Resolution WHA66.12: anthrax, brucellosis, and bovine tuberculosis. These three were highlighted as grave zoonotic diseases in the early years of the WHO, with ECZs specifically established to discuss their control in 1948–1950, and are major zoonoses, impacting human and animal health in developing economies.

The passing of Resolution WHA66.12 in May 2013 is seen as a significant step in the right direction towards increased advocacy for the NTDs. The Resolution raises awareness of the potential for integrated control and surveillance systems that tackle a number of coendemic diseases simultaneously under a One Health approach. Viewing control of zoonotic diseases in terms of a joint approach to improve human and animal health and production in endemic countries makes sense both from a development perspective and also in terms of food safety and security. There is acknowledgment that by addressing the underlying systemic issues of disease surveillance and response for the NZDs, the capacity for detection of emerging zoonoses will also be strengthened. The promotion of a One Health approach towards surveillance and control of NZDs in developing countries is also embedded within the NTD research agenda, being seen as “interdisciplinary, participatory, and integrated with prevention and control needs” [25].

**Box 1. Key Learning Points**

- Global health priorities are many and varied; however, unlike high profile diseases such as HIV/AIDS, malaria, and H5N1 avian influenza, the neglected tropical diseases have historically been “forgotten” in terms of funding and collective action for control.
- The passing of the recent World Health Assembly Resolution WHA66.12 is a key step in the advocacy process for the neglected tropical diseases, acknowledging the research and advocacy efforts of those involved in their control.
- Five of the eight neglected zoonotic diseases—cysticercosis, rabies, echinococcus, human African trypanosomiasis, and leishmaniasis—are included in the list of NTDs identified by Resolution WHA66.12. However, anthrax, bovine tuberculosis, and brucellosis are still lacking the high-level advocacy required to mobilise political support for their control in endemic countries.

**Box 2. Key Papers in the Field**

- Hotez PJ, Molyneux DH, Fenwick A, Kumaresan J, Sachs SE, et al. (2007) Control of neglected tropical diseases. N Engl J Med 357: 1018–1027. doi:10.1056/NEJMra064142
- Molyneux DH (2008) Combating the “other diseases” of MDG 6: Changing the paradigm to achieve equity and poverty reduction? Trans R Soc Trop Med Hyg 102: 509–519. doi:10.1016/j.trstmh.2008.02.024
- Maudlin I, Eisler MC, Welburn SC (2009) Neglected and endemic zoonoses. Phil Trans R Soc B 364: 2777–2787. doi: 10.1098/rstb.2009.0067
- Okello AL, Gibbs EPJ, Vandersmissen A, Welburn SC (2011) One Health and the neglected zoonoses: turning rhetoric into reality. Vet Rec 169: 281–285. doi: 10.1136/vr.d5378
- WHO (2012) Accelerating work to overcome the global impact of neglected tropical diseases: A roadmap for implementation. Geneva: World Health Organization. Available: http://www.who.int/neglected_diseases/NTD_RoadMap_2012_Fullversion.pdf. Accessed 14 March 2014.
